# Clonal spread and environmental persistence of carbapenem-resistant high-risk *Pseudomonas aeruginosa* in critical-care units of a Chilean national referral center for burn and trauma patients (2022)

**DOI:** 10.15698/mic2026.07.883

**Published:** 2026-07-20

**Authors:** Camila Ibarra, Gustavo Araya, Alhejandra Alvarez, Leonardo Enríquez, Carolina Arellano, Fabricio Alarcón, Constanza Fernández, Claudio Vargas, Rodrigo Vera, Roberto M. Vidal

**Affiliations:** 1 Hospital de Urgencia Asistencia Pública, Chile; 2 Núcleo Interdisciplinario de Microbiología, Instituto de Ciencias Biomédicas (ICBM), Facultad de Medicina, Universidad de Chile, Chile

**Keywords:** *Pseudomonas aeruginosa*, carbapenem resistance, clonal dissemination, environmental persistence, Whole-genome sequencing

## Abstract

*Pseudomonas aeruginosa* is an opportunistic nosocomial pathogen ranked by the World Health Organization (WHO) as a high priority for research and the development of new antimicrobial therapies. To characterize carbapenem-resistant *P. aeruginosa* (CRPA) isolated from patients at the Hospital de Urgencia Asistencia Pública (HUAP), Chile in 2022, and to evaluate clonal diversity, resistance mechanisms, virulence factors, and clinical associations. We analyzed 126 clinical CRPA isolates using PFGE, PCR screening of resistance and virulence genes, and whole-genome sequencing of 18 representative strains. Sequence types (STs), resistomes, and virulomes were identified, and their associations with severity, length of hospital stay, cost, and patient outcomes were evaluated. Seventy-four percent of isolates originated from critical care units, predominantly from respiratory and tissue samples. PFGE revealed 25 pulsotypes, with L and Y being the predominant types. Overall, 67% of isolates were XDR and 1% PDR. Carbapenemase genes were absent in 115 isolates, while *blaVIM* and *blaKPC* were detected in 8 and 3 isolates, respectively. The *exoS*^+^/*exoU*^+^ genotype was identified in 6 isolates; whole-genome sequencing revealed eight distinct STs, including previously described high-risk clones ST654, ST395, and ST274. A resistome analysis revealed diverse aminoglycoside and β -lactam resistance determinants, while a virulome analysis confirmed the presence of *exoS*^+^/*exoU*^+^ in two sequenced isolates. Carbapenem resistance was significantly associated with prolonged hospitalization (median 84 vs. 39 days), greater clinical severity, and substantially higher healthcare resource utilization, reflected by increased DRG (Diagnosis-Related Group) weights, compared with carbapenem-susceptible controls. Our findings highlight the circulation of high-risk *P. aeruginosa* clones in Chile and underscore the importance of molecular epidemiology in guiding infection control, optimizing antimicrobial therapy, and mitigating the clinical and economic burden of CRPA.

## INTRODUCTION

*Pseudomonas aeruginosa* is a Gram-negative bacillus (1–5 μ m long, 0.5–1 μ m wide), strictly aerobic, oxidase-positive, non-fermentative, and motile via a single polar flagellum 1. It is ubiquitous, inhabiting aquatic and soil environments, as well as the tissues of plants, animals, and humans 2. Hospitals are a favorable niche due to their ability to survive in nutrient-poor aqueous environments and form biofilms on surfaces such as sinks, faucets, showers, disinfectants, and medical devices 3. Transmission occurs via environmental sources or cross-transmission through the contaminated hands of healthcare personnel. These routes constitute key risk factors for *P. aeruginosa* colonization and infection, along with intensive care unit (ICU) hospitalization, prior antibiotic exposure, and the use of invasive devices 4, 5.

As an opportunistic pathogen, *P. aeruginosa* causes infections in immunocompromised patients, those with cystic fibrosis, and hospitalized individuals, commonly associated with ventilator-associated pneumonia (VAP), urinary tract infections, surgical site infections, burn wounds, and bloodstream infections 6. In acute infections, particularly VAP, colonization of endotracheal tubes and biofilm formation are critical. Conversely, in cystic fibrosis, impaired mucus clearance and biofilm persistence promote the development of chronic disease 7, 8.

Virulence relies on a broad arsenal including surface structures (type IV pili, polar flagellum, lipopolysaccharide (LPS)) 9; the type III secretion system (ExoU, ExoS, ExoT, ExoY) 10, 11; extracellular enzymes (exotoxin A, elastases, lipases, and phospholipases) 9, 12; and biofilm formation mediated by exopolysaccharides such as alginate, Psl, and Pel 13, 14.

Antibiotic resistance is mediated by intrinsic and acquired mechanisms. Intrinsic mechanisms include reduced permeability (OprF, OprD), efflux pumps (Mex systems) and chromosomal AmpC 15–17. Acquired resistance arises from mutations (e.g., OprD loss, *gyrA*, *ampC*), or horizontal transfer of carbapenemase genes (*blaGES, blaIMP, blaVIM, blaKPC, blaNDM, blaOXA*) 15, 18. Carbapenem resistance can result from porin loss, efflux pump overexpression, AmpC derepression, or carbapenemase acquisition 19–21. These mechanisms limit therapy, leaving ceftazidime, cefepime, piperacillin/tazobactam, carbapenems, fluoroquinolones, and aminoglycosides as options, with colistin and novel agents (ceftazidime/avibactam, ceftolozane/tazobactam) reserved for multidrug-resistant isolates 22.

*P. aeruginosa* has been designated by the WHO as a high-priority pathogen due to MDR, XDR, and PDR profiles 23. Globally, high-risk clones (HRCs) such as ST235, ST111, ST175, and ST654 have spread widely and are associated with high mortality 24. In Latin America, ST235, ST111, ST244, ST277, ST357, and ST308 have been reported 24–27. In Chile, PubMLST lists 14 sequence types up to 2025, including ST654 28. In addition, other sequence types, such as ST357 and ST309, have also been identified in the country by independent research groups 29–31.

**Figure 1:**
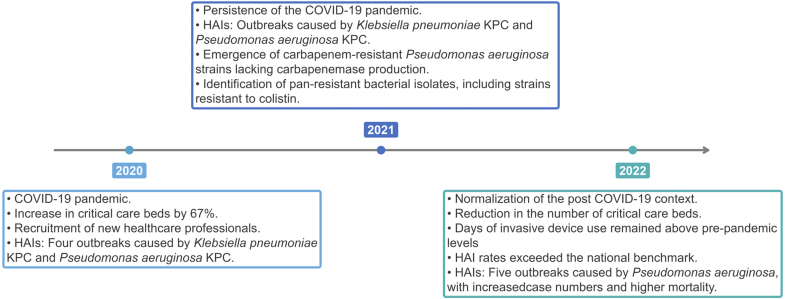
Local epidemiological data, HUAP 2020–2022. Timeline of local epidemiological trends at HUAP from 2020 to 2022. The impact of the COVID-19 pandemic, expansion of critical care capacity, recruitment of additional healthcare personnel, and other contributing factors promoted the emergence and proliferation of multidrug-resistant bacteria. HAIs: Healthcare-associated infections. KPC: *Klebsiella pneumoniae* carbapenemase.

Healthcare-associated infections (HAIs) remain a significant concern. In Chile, *P. aeruginosa* accounted for 25% of VAP cases in 2022, with carbapenem resistance rates of ∼ 48% (34,35). Hospital de Urgencia Asistencia Pública (HUAP), a national referral center for burn and trauma patients, has a total capacity of 294 beds, including 97 critical care beds; this number increased from 57 to 97 during the COVID-19 pandemic. In 2022, a total of 10,682 patients were hospitalized, of whom 3,513 were admitted to critical care units (CCU) and 951 required invasive mechanical ventilation 32. At HUAP, outbreaks caused by carbapenem-resistant *P. aeruginosa* including carbapenemase-producing isolates harboring the *blaKPC* gene, as well as CRPA (carbapenem-resistant *P. aeruginosa*) without detectable carbapenemase genes, have been documented 32. In 2022, despite the post-pandemic reduction in ICU bed capacity, *P. aeruginosa* was responsible for five outbreaks (52 infections, 17 deaths), was isolated from 798 cultures obtained from 297 patients, and showed 55% resistance to imipenem and meropenem 32 (Figure 1). Compared with previous years, the number of positive *P. aeruginosa* cultures increased substantially beginning in 2020 and remained elevated through 2022. This trend coincided with the expansion of critical-care capacity during the COVID-19 pandemic, including a marked increase in intensive-care beds and mechanically ventilated patients (Table S1, Annex 1). Given this epidemiological scenario and the emergence of high-risk clones, molecular and epidemiological characterization of CRPA isolates from HUAP is essential to assess their association with hospital stay, disease severity, and patient outcomes.

## RESULTS

A total of 126 non-duplicate carbapenem-resistant *Pseudomonas aeruginosa* (CRPA) clinical isolates were collected during 2022 at the HUAP, a national referral center for critical, trauma, and burn patients in Chile. The majority of isolates originated from CCU (93 isolates; 74%), followed by 16 isolates from the operating room (OR) (12,7%), 10 from medium care units (MCU) (7,9%), 5 from the emergency department (ED) (4%), and 2 from other departments (1,6%). These findings reflect the significant burden of infections in intensive care settings, with de majority of these cases originating from the burn unit (54 isolates, 60%). Respiratory tract specimens (47 isolates, 37%) were the predominant source, followed by wound and tissue samples (38 isolates, 30%), highlighting the diverse clinical manifestations of CRPA infections (Figure S1, Annex 1).

**Figure 2:**
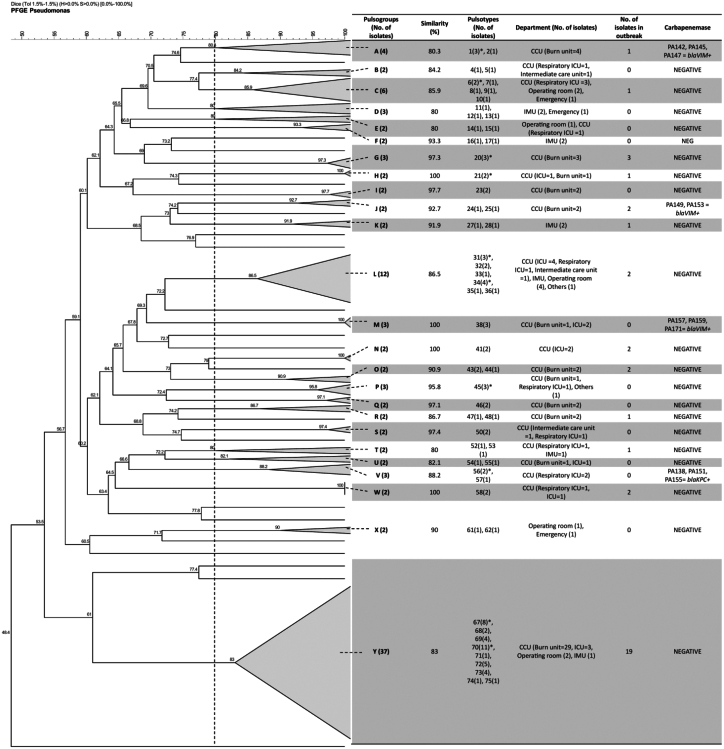
Dendrogram of PFGE analysis using the SpeI restriction enzyme . Twenty-five pulsogroups (A-Y) are shown. The figure illustrates the pulsogroups, the percentage of similarity among isolates, pulsotypes, and the clinical services from which the samples were obtained, as well as the number of isolates involved in hospital-defined outbreaks and isolates with the carbapenemase gene. Outbreak-associated isolates correspond to the subset of isolates epidemiologically linked to hospital-defined outbreak events during 2022. An asterisk * indicates the pulsotypes to which the isolates were selected for sequencing. CCU: Critical care units, ICU: Intensive care unit, IMU: Intermediate care unit.

Molecular typing by pulsed-field gel electrophoresis (PFGE) revealed considerable genetic diversity. Clonality results for the 126 isolates identified a total of 25 pulsogroups, categorized from A to Y, with a similarity percentage greater than 80%. Additionally, 76 pulsotypes showed a similarity of 95% or higher, as shown in Figure 2. Despite this heterogeneity, pulsogroups L and Y predominated, suggesting the occurrence of clonal clusters circulating within critical care units. The chronological mapping of isolate recovery across hospital facilities provided additional epidemiological insights. Specifically, ST3974 (pulsogroup Y; pulsotypes 67 and 70), initially detected during a previous outbreak in the old hospital building in 2021, was subsequently identified in patients hospitalized in the newly inaugurated facility during 2022, suggesting persistence and inter-facility dissemination of this lineage. This observation highlights how specific CRPA lineages can adapt to diverse clinical environments and persist despite changes in infrastructure.

**Figure 3:**
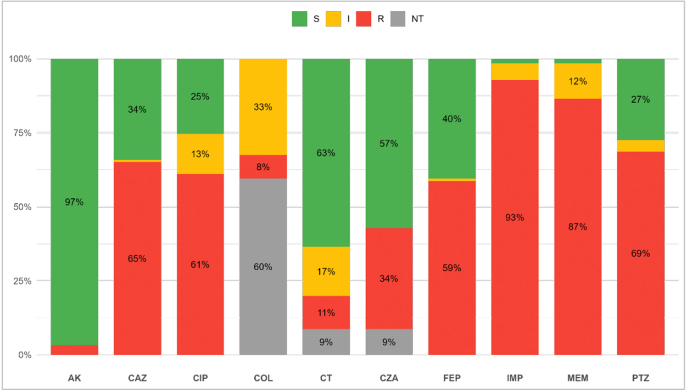
Antibiotic susceptibility results . Antimicrobial susceptibility testing performed for the 126 isolates by automated broth microdilution using the Vitek 2.0 Compact system (bioMérieux). CAZ: Ceftazidime; FEP: Cefepime; IPM: Imipenem; MEM: Meropenem; PTZ: Piperacillin/tazobactam; AK: Amikacin; CIP: Ciprofloxacin; COL: Colistin; CZA: Ceftazidime/avibactam; CT: Ceftolozane/tazobactam. S: Susceptible; R: Resistant; I: Intermediate. Interpretation according to CLSI 2024 breakpoints. N/T: Not tested.

Antimicrobial susceptibility testing revealed strikingly high levels of resistance. Antimicrobial susceptibility profiles were determined for all 126 CRPA isolates included in the study. Based on established definitions 33, 67% of isolates were classified as extensively drug-resistant (XDR) and 1% as pandrug-resistant (PDR). Carbapenem resistance was nearly universal, with 93% and 87% of isolates resistant to imipenem and meropenem, respectively. Other β -lactams displayed limited activity: the susceptibility rates to ceftazidime and cefepime were 34% and 40%, respectively, while piperacillin/tazobactam was active against only 22% of the isolates. Fluoroquinolone resistance was widespread, with ciprofloxacin susceptibility reduced to 25%. Polymyxin susceptibility testing showed variable results, with 33% of isolates exhibiting intermediate susceptibility and only 8% being fully susceptible to colistin. In contrast, amikacin remained the most effective agent, with 94% of isolates susceptible, confirming aminoglycosides as one of the few remaining therapeutic options. Among newer β -lactam/ β -lactamase inhibitor combinations, susceptibility rates were moderate: 57% for ceftazidime/avibactam and 63% for ceftolozane/tazobactam (Figure 3). These data underscore the limited therapeutic arsenal available for treating CRPA at HUAP.

Carbapenemase gene screening showed that resistance was not primarily driven by carbapenemase production. 115/126 isolates tested negative for all major carbapenemase families, highlighting the importance of alternative resistance mechanisms. Nevertheless, carbapenemases were detected in 11 isolates: 8 carried Verona integron-encoded metallo- β -lactamases (VIM) and 3 carried *Klebsiella pneumoniae* carbapenemases (KPC). The coexistence of isolates with and without carbapenemase genes illustrates the complex genetic background of carbapenem resistance in this setting.

**Table 1:** Percentages and frequencies of the different exoenzyme genotypes (A) and virulence gene profiles across the studied isolates (B).

**A**		
**Exoenzymes**	**N**	**%**
*exoY* ${}^{+}$ *, exoT* ${}^{+}$ *, exoS* ${}^{+}$	88	70%
*exoY* ${}^{+}$ *, exoT* ${}^{+}$ *, exoU* ${}^{+}$	21	17%
*exoT* ${}^{+}$ *, exoS* ${}^{+}$	8	6%
*exoY* ${}^{+}$ *, exoT* ${}^{+}$ *, exoS* ${}^{+}$ *, exoU* ${}^{+}$	6	5%
*exoY* ${}^{+}$ *, exoT* ${}^{+}$	1	1%
*exoT* ${}^{+}$ *, exoU* ${}^{+}$	1	1%
-	1	1%
Total	126	100%

**B**		
**Virulence factors**	**No. of isolates**	**%**
*exoT*	125	99%
*exoY*	116	92%
*exoS*	102	81%
*exoU*	28	22%

Virulence profiling by PCR focused on type III secretion system effector genes. The majority of isolates carried *exoS*, while *exoU* was less frequent. Notably, six isolates were positive for both *exoS* and *exoU*, a genotype combination previously considered mutually exclusive 10. These *exoS^+^/exoU^+^* isolates are of particular concern, as they have been described as hypervirulent and associated with severe infections in various geographic regions. ExoU exerts a potent cytotoxic effect on host cells and has been associated with increased mortality 34, whereas ExoS promotes invasive phenotypes and host-cell apoptosis 35, 36. Their detection in this cohort suggests the circulation of highly virulent CRPA lineages within this tertiary-care hospital setting (Table 1 and Figure S2). Among the remaining virulence factors, all 126 isolates were positive for the *algD, plcH*, *plcN*, and *lasB* genes; however, the *toxA* exotoxin gene was detected in only 91% of the isolates.

**Figure 4:**
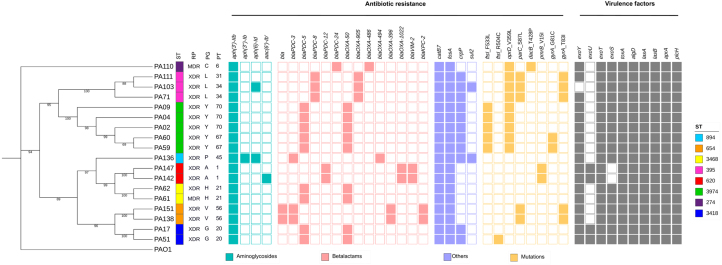
Phylogenetic tree and distribution of gene presence/absence among the 18 sequenced strains based on the alignment generated by Snippy-core (86,760 informative SNPs). The sequence type (ST), resistance phenotype (RP), pulsogroups (PG), pulsotype (PT), and antibiotic resistance genes grouped by antibiotic class (aminoglycosides, β -lactams, and others), as well as antibiotic resistance acquired through point mutations, are shown. Finally, representative virulence factor genes detected are displayed. Filled squares indicate the presence of a given gene or mutation, whereas empty squares indicate its absence. Figure created using iTOL.

Whole-genome sequencing (WGS) was performed on 18 representative isolates selected from the PFGE dendrogram to further characterize their genomic backgrounds. Selection was based on capturing isolate diversity by including one representative clone from pulsogroups comprising more than two isolates, while accounting for variability in clinical settings, sample origin, antimicrobial resistance phenotypes, and carbapenemase gene carriage. Sequence typing revealed eight distinct sequence types (STs), including the globally recognized high-risk clones ST654, ST395, and ST274, previously described by Jesús Del Barrio-Tofiño 2020 24. The presence of these clones, associated with multidrug resistance and high transmissibility, emphasizes their clinical importance. A resistome analysis revealed a diverse array of aminoglycoside-modifying enzyme genes, including *aac*, *aph*, and *ant* variants, as well as multiple β -lactam resistance determinants, which collectively explain the broad phenotypic resistance patterns. Virulome profiling confirmed the coexistence of *exoS* and *exoU* in two sequenced isolates, validating PCR findings. These results establish clear connections between local CRPA lineages and high-risk international clones, reinforcing the importance of genomic surveillance (Figure 4).

**Table 2:** Statistical analysis among the three defined groups.

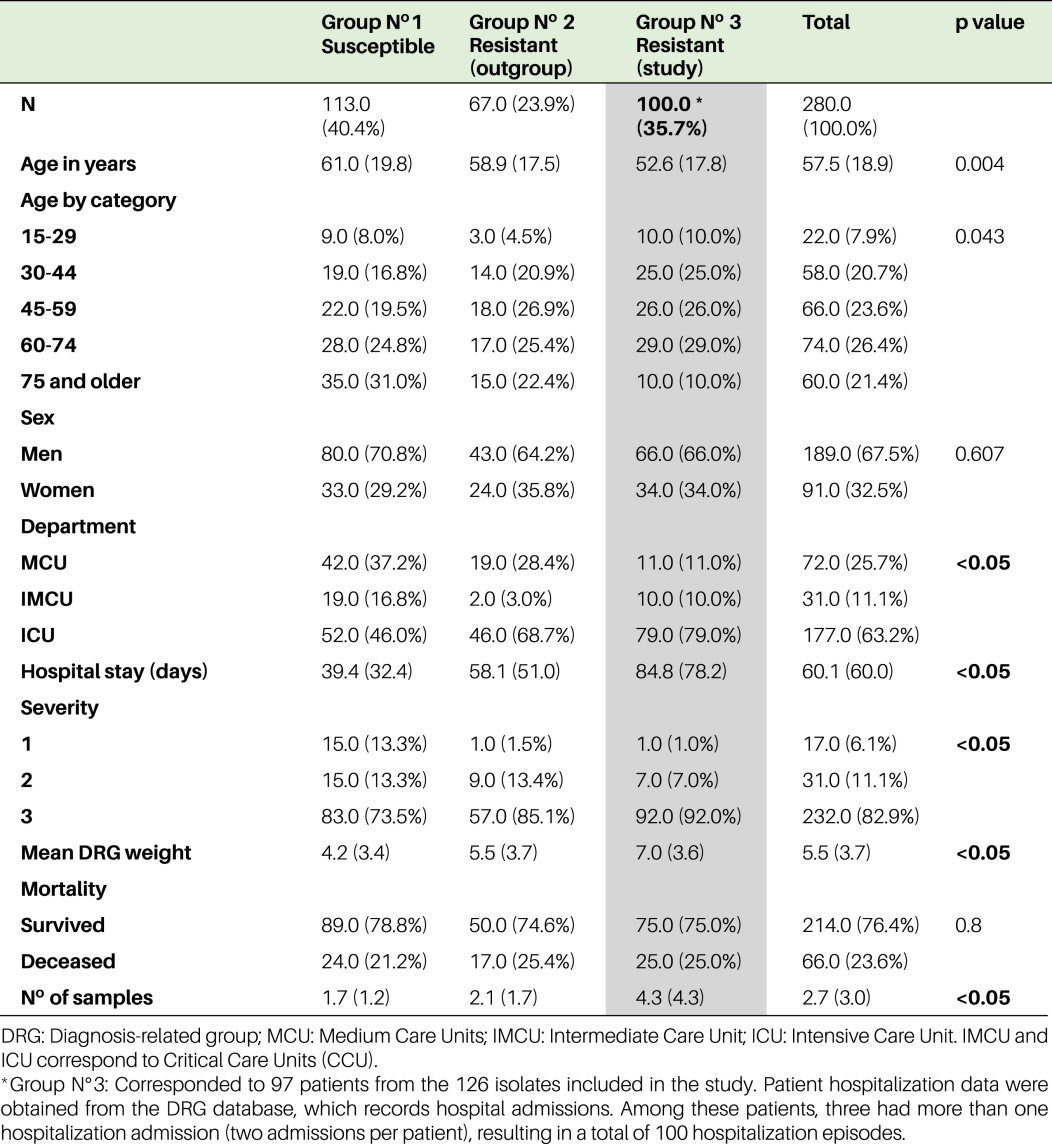

The integration of molecular data with clinical and epidemiological outcomes revealed significant associations. Most CRPA patients were hospitalized in critical care units (79%), whereas carbapenem-susceptible *P. aeruginosa* patients were more frequently distributed across intermediate care units (IMU) (37.2%) (Table 2). Consistently, most CRPA isolates were recovered from critical care units (93 isolates; 73.8%), followed by operating rooms (16 isolates; 12.7%), IMU (10 isolates; 7.9%), emergency department (5 isolates; 4.0%), and other hospital departments (2 isolates; 1.6%) (Figure S1). In 2022, *P. aeruginosa* was also involved in five epidemiologically documented hospital outbreaks at HUAP, resulting in 52 infections and 17 associated deaths, according to institutional surveillance records. Patients infected with CRPA had a significantly longer hospital stay, with a median of 84 days compared to 39 days for those infected with carbapenem-susceptible *P. aeruginosa*. Clinical severity was higher among CRPA patients, as assessed by the Severity of Illness (SOI) classification system derived from DRG (diagnosis-related groups) records. Specifically, 92% of patients in the CRPA group were classified within the highest severity category, compared with 73.5% of patients infected with carbapenem-susceptible *P. aeruginosa*. Resource utilization was substantially greater for CRPA cases, with an average DRG weight of 5.5 compared to 4.2 in controls, indicating a higher complexity of care. CRPA infections were associated with substantially greater healthcare resource utilization, as reflected by significantly higher DRG weights than carbapenem-susceptible infections. In-hospital mortality rates were comparable across groups, with mortality observed in 21.2% of carbapenem-susceptible patients, 25.4% of the resistant outgroup, and 25.0% of CRPA study patients ( p=0.8 ). However, CRPA patients presented a higher number of positive cultures per patient (2.7 vs. 1.7), suggesting persistent colonization or recurrent infection (Table 2). Some patients presented multiple *P. aeruginosa* isolates during hospitalization, including isolates with distinct pulsotypes, resistance profiles, and virulence genotypes, as summarized in Supplementary Table S2.

Altogether, the results demonstrate that CRPA circulating in HUAP during 2022 exhibited both high clonal diversity and evidence of clonal persistence, including transmission across different hospital infrastructures. The isolates exhibited extensive resistance profiles, primarily driven by non-carbapenemase mechanisms, with sporadic detection of *blaVIM* and *blaKPC* carbapenemases. Virulence profiling revealed *exoS*^+^/*exoU*^+^ genotypes in a subset of isolates, while WGS identified multiple high-risk sequence types also reported in other regions worldwide. Clinically, CRPA infections were associated with prolonged hospital stays, higher severity scores, and greater healthcare resource utilization, consolidating their role as a critical challenge in nosocomial infection management. The detection of a clone migrating from the old hospital building to the new one highlights the adaptability of CRPA lineages and the urgent need for genomic epidemiology to monitor and prevent intra-hospital dissemination.

## DISCUSSION

This study presents an integrated molecular and epidemiological overview of carbapenem-resistant *Pseudomonas aeruginosa* (CRPA) at HUAP in 2022, examining the connections between clonal structure, resistance and virulence determinants, and patient-level impact. Three features characterize the local landscape. First, the burden of disease was concentrated in critical-care settings, consistent with recognized risk factors: ICU stay and prior antimicrobial exposure 37. At HUAP, 74% of isolates originated from critical units, predominantly respiratory and tissue specimens, reflecting syndromes typical of ventilated and severely ill patients. The major burns cohort is locally significant: as a national referral center, HUAP cares for patients in whom *P. aeruginosa* commonly colonizes and infects burn wounds, with time-dependent tissue colonization reported in the literature 38, 39. Second, pulsed-field gel electrophoresis (PFGE) revealed substantial diversity—25 pulsogroups (A–Y)—alongside two dominant clusters (L, Y). Using >80% similarity to define pulsogroups and ≥ 95% to define indistinguishable pulsotypes, large PFGE groups centered on L and particularly Y, concentrating cases in critical care and the burns service (Figure 2). One hospital-defined outbreak mapped largely to Y, whereas others spanned additional clusters, indicating that institutional events can reflect amplification of a successful lineage while distinct clones circulate contemporaneously. This pattern aligns with the ecology of *P. aeruginosa* in complex care environments, where resilient wet reservoirs support multiple lineages and device-intensive workflows facilitate clonal spread. Third, resistance profiles were severe: 67% of isolates were XDR, and 1% PDR. Carbapenem resistance was nearly universal, due to the inclusion criteria; carbapenemase production was detected in a minority of isolates (VIM in 8, KPC in 3), underscoring alternative resistance mechanisms. Phenotypically, amikacin retained the highest activity; newer β -lactam/ β -lactamase inhibitor combinations showed only partial activity; and most available colistin minimum inhibitory concentrations (MICs) fell in intermediate or resistant categories, since the CLSI does not define a susceptible breakpoint for this antibiotic. Applying difficult-to-treat resistance (DTR) criteria identified nearly half of isolates as DTR, highlighting constrained options and the potential toxicity of salvage regimens 40. Genotypically, multiple mechanisms converged. Diverse *blaPDC* (AmpC) and *blaOXA* variants across isolates. In non-carbapenemase CRPA, *oprD* alterations (e.g., OprD_V359L) plausibly explained imipenem resistance when combined with AmpC; *ftsI* (PBP3) substitutions (F533L within ST3974; R504C in ST3418) associated with decreased susceptibility to extended-spectrum β -lactams and newer β -lactam/ β -lactamase inhibitor agents. Quinolone resistance-determining region (QRDR) changes in *gyrA*/*parC* mapped to high-level fluoroquinolone resistance. Finally, *pmrB_V15I* was detected in two *blaVIM-2*-positive ST620 isolates—classically linked to colistin resistance, though phenotypic confirmation was limited by testing scope. Taken together, these findings support a multifactorial resistance architecture that varies across different lineages. Virulence profiling added an important dimension. While T3SS components and ancillary virulence genes were broadly present, six isolates carried the *exoS*^+^/*exoU*^+^ genotype—a combination associated with hypervirulence in experimental and clinical reports 35. WGS confirmed the presence of *exoS^+^/exoU^+^* in two sequenced isolates, reinforcing PCR findings and indicating that particular HUAP lineages combine difficult resistance profiles with potent virulence repertoires—a concerning configuration in units with prolonged device use.

At clonal resolution, WGS identified eight sequence types, including previously described high-risk clones (HRCs) ST654, ST395, and ST274, which linked local epidemiology to global CRPA lineages. Locally, ST654 carried *blaKPC-2* in two isolates, as previously reported in Chile 31. In contrast, ST395 (globally disseminated) and ST274 (increasingly recognized as HRC) lacked carbapenemases, again pointing to non-carbapenemase mechanisms. Non-HRC singletons (ST894, ST3468, ST620, ST3974, ST3418) nonetheless accumulated clinically significant determinants; notably, ST3974 was detected across multiple services, and temporal mapping showed one ST introduced in older facilities subsequently recovered in patients from the new hospital building, evidencing persistence and intra-institutional spread.

Additionally, ST3418 is worth highlighting, as this ST has recently been reported in China as a hypervirulent lineage associated with the *exoS*^+^/*exoU*^+^ genotype 41.

Clinical and economic signals were robust. Compared with carbapenem-susceptible *P. aeruginosa*, CRPA infections were associated with notably longer hospitalizations (median ∼ 84 vs. ∼ 39 days), higher severity categories, greater resource utilization (higher DRG weight), and approximately threefold higher costs. Mortality did not differ significantly, but CRPA patients had more positive cultures per patient, consistent with persistence or recurrence. These findings extend international experience by linking genomic epidemiology to patient-level burden in a national burns/critical-care referral context, underscoring the need for targeted infection-prevention measures.

### Programmatic implications

Given that most CRPA were non-carbapenemase producers, laboratory and IPC (Infection Prevention and Control) strategies had to go beyond carbapenemase screening to incorporate markers of OprD disruption, AmpC variation, and DTR phenotypes. Combining PFGE/WGS with granular unit-level mapping can localize transmission routes—large clusters (e.g., pulsogroup X) spanning multiple critical services—informing interventions (e.g., respiratory-circuit handling, targeted environmental sampling of wet reservoirs, device reprocessing audits) commensurate with the observed spread. Antimicrobial stewardship should prioritize the judicious use of aminoglycosides when appropriate, newer β -lactam/ β -lactamase inhibitors, and early infectious-disease consultation for DTR scenarios.

### Limitations

This single-center study prioritized 18 representative isolates for WGS. While sufficient to anchor clonal and mechanistic inferences, broader sequencing would refine lineage attribution. Efflux-regulator mutations were not assessed, potentially underestimating their contribution. Colistin phenotyping was limited to a subset, complicating genotype–phenotype correlation. As an observational analysis, residual confounding in clinical associations cannot be excluded. These constraints do not alter the central conclusions but rather highlight areas for further expansion. Future work should extend WGS to the full collection, integrate efflux-regulator analyses, and add systematic environmental sampling to resolve reservoir contributions. Embedding rapid genomic analytics into routine IPC could shorten the interval between detection and control, particularly during unit transitions or infrastructure changes.

## CONCLUSIONS

CRPA at HUAP in 2022 was primarily observed in critical care settings, with a predominant focus on respiratory and tissue involvement. Isolates exhibited clonal clustering, with two dominant PFGE groups, and were documented to have spread across services, including persistence from older facilities into the new building. They showed severe resistance (67% XDR; 1% PDR) largely without carbapenemases (*blaVIM* in 8; *blaKPC* in 3), driven by combinations of AmpC/OXA variants, OprD disruption, QRDR, and *ftsI*/*dacB* changes. A subset carried *exoS*^+^/ *exoU*^+^ (confirmed by WGS), and infections were associated with prolonged hospitalization, higher severity, and substantially increased costs. Local HUAP isolates (ST654, ST395, and ST274) correspond to sequence types previously described as globally disseminated high-risk clones, emphasizing the relevance of these lineages within the international epidemiology of *P. aeruginosa*. These findings support genomics-informed IPC, focusing on critical-care workflows and wet-reservoir control, as well as laboratory algorithms that capture non-carbapenemase resistance, and stewardship that is attentive to DTR phenotypes. Scaling WGS to all isolates and coupling it with environmental surveillance should help pinpoint reservoirs and interrupt transmission in this high-risk setting.

## MATERIALS AND METHODS

### Bacterial isolates

We studied 126 CRPA isolates from 97 individual patients, obtained at the HUAP Clinical Microbiology Laboratory in 2022. Because three patients had more than one hospital admission during 2022, the epidemiological analysis included 100 hospitalization records obtained from the institutional DRG database. All comparison groups comprised patients hospitalized in 2022, and the resistant outgroup included carbapenem-resistant isolates that were not recoverable or unavailable for molecular characterization due to the study’s retrospective design. Isolates were obtained from patients in critical care units (CCU), intermediate care units (IMU), operating rooms, the emergency department, and other services. Samples were obtained from five infection sites: respiratory tract, blood, urine, sterile body fluids, and tissues. Only isolates with MICs to imipenem or meropenem classified as intermediate (4 μ g/mL) or resistant ( ≥ 8 μ g/mL) according to the CLSI 2024 guidelines were included.

### Identification and antimicrobial susceptibility

Species identification and susceptibility were performed with the Vitek 2.0 Compact system (bioMérieux). Susceptibility to ceftazidime, cefepime, imipenem, meropenem, piperacillin/tazobactam, amikacin, and ciprofloxacin was tested by automated broth microdilution. Additional testing included ceftazidime/avibactam, ceftolozane/tazobactam, and colistin (the latter tested by manual broth microdilution, LiofilChem). Results were interpreted according to CLSI 2024 breakpoints. Carbapenemase production was detected with O.K.N.V.I RESIST-5 (Coris BioConcept). Negative cases were confirmed by the in-house CARBA-NP test 42. Strains were classified as MDR, XDR, or PDR according to international criteria 33.

### Pulsed-field gel electrophoresis (PFGE)

PFGE was performed on all isolates following CDC protocols 43. DNA plugs were digested with *SpeI* (ThermoFisher) and separated at 6 V/cm for 21 hours in a CHEF-DR III (Bio-Rad) apparatus. Banding patterns were visualized with ethidium bromide and analyzed with GelCompar II (Applied Maths). Similarity was calculated using Dice coefficients, and dendrograms were generated with the UPGMA method. *Salmonella enterica* Braenderup ATCC was used as a reference. Clusters were defined as pulsogroups at a similarity level of >80% and pulsotypes at a similarity level of ≥ 95%.

### Resistance genotype and virulence factors

DNA was extracted with the Wizard Genomic DNA Purification Kit (Promega). Multiplex PCRs were used to amplify carbapenemase genes (*blaKPC, blaNDM, blaOXA-48, blaIMP*, and *blaVIM*), following the methods described by Candan & Aksöz (2015) and Poirel et al. (2011) 44, 45. Control strains included KPN BAA-1705, BAA-2524, UCO-361, UCO-143, and PA-HUAP VIM. Primers and protocols are detailed in **Tables S3 and S4 (Annex 1)**. Virulence genes (*exoS, exoT, exoU, exoY, algD, lasB, toxA, plcH, plcN*) were screened using published multiplex PCR protocols 46–50. Controls included *P. aeruginosa* ATCC 27853 and PA14. Details are provided in **Tables S4 and S5 (Annex 1)**.

### Whole-genome sequencing, MLST, resistome, and virulome

Eighteen representative isolates were selected from PFGE clusters. DNA was extracted using the Wizard Kit (Promega) and sequenced at MicrobesNG (University of Birmingham, UK) on an Illumina MiSeq/HiSeq platform. Assemblies were generated with SPAdes v3.10 51 and evaluated with QUAST v4.6.3 52. Resistome and virulome analyses were performed in Galaxy with AMRFinder 53, ABRicate 54, and VFDB 55. Sequence types were determined with PubMLST 28. A minimum spanning tree was built using PHYLOViZ 56 with goeBURST. Genomes were annotated using Prokka 57, compared with Roary 58, and aligned with Snippy for SNP analysis 59. Phylogenies were inferred with IQ-TREE 60 and visualized with iTOL 61. Contigs shorter than 200 nt were removed, and sequences were deposited at GenBank under Bioproject PRJNA1348171.

### Epidemiological data

Clinical and microbiological data were retrieved from the HUAP diagnosis-related group (DRG) system and KernMic system (Biomerieux). Patients were classified into three groups according to antimicrobial susceptibility profile: (1) carbapenem-susceptible P. aeruginosa isolates (control group), comprising patients whose isolates remained susceptible to imipenem and meropenem throughout hospitalization; (2) carbapenem-resistant outgroup isolates excluded from molecular analysis because isolates were unavailable or could not be recovered from cryostorage due to the retrospective nature of the study. However, their associated clinical and epidemiological data were retained for comparative analysis in Table 2; and (3) carbapenem-resistant isolates included in the present molecular epidemiology analysis. Microbiological records were linked with hospital discharge and administrative datasets using unique patient and hospitalization identifiers. Hospital resource utilization and clinical complexity were obtained from DRG system. DRG weight represents the expected healthcare resource utilization and clinical complexity associated with a given hospitalization relative to the average cost of hospitalized patients. This hospital management system is based on discharge records and enables classification of patients by clinical characteristics and healthcare resource use during hospitalization. Variables incorporate into the DRG system include age, sex, diagnosis, procedures performed, comorbilities, length of stay, and discharge status among others. Clinical severity was categorized using the SOI index, which classifies patients into four levels (0 – 3) of clinical severity based on primary diagnosis, comorbidities, therapeutic interventions, level of dependency, and response to therapy. In which level 0 corresponds to minor severity (ambulatory patients), level 1 to moderate severity, level 2 to major severity, and level 3 to extreme severity 62.

### Statistical analysis

Statistical analyses were performed using STATA v18. Categorical variables were compared with Pearson’s chi-square or Fisher’s exact tests, while continuous variables were compared using parametric or non-parametric tests according to data distribution. p < 0.05 was considered statistically significant.

### Ethics and Consent to Participate

The use of clinical and microbiological data was approved by the Scientific Ethics Committee of the Metropolitan Central Health Service (SSMC) and the HUAP authorities (Supplementary material, Annex 2, File N ${}^{\circ }$ 60-10 N ${}^{\circ }$ 350/2023). The Ethics Committee determined that individual informed consent was not required, as the study was retrospective and based solely on anonymized clinical and microbiological data, with no direct patient contact, in accordance with institutional regulations and national ethical guidelines.

## AUTHORS CONTRIBUTIONS

Conceptualization and experimental design: CI, CV, RV, and RMV; Methodology: CI, CV, and RMV; Data acquisition: CI, CA, FA, CF; Bioinformatics Pipelines: CI, GA and LE; Data analysis, visualization and interpretation: CI, GA, AA, LE, CV and RMV; Resources and Funding Acquisition: RMV; Supervision: RV and RMV; Writing original draft: CI and RMV.; Review: CI, CV, RV and RMV. Final Edition: CI, RV and RMV.

## SUPPLEMENTAL MATERIAL

All supplemental data for this article are available online at http://www.microbialcell.com/researcharticles/2026a-ibarra-microbial-cell/.

.

## CONFLICT OF INTEREST

The authors declare that they have no competing interests.

## ABBREVIATIONS

CCU – critical care unit

CRPA – carbapenem-resistant *P. aeruginosa*

DRG – diagnosis-related group

HAIs – healthcare-associated infections

HRCs – high-risk clones

HUAP – Hospital de Urgencia Asistencia Pública

ICU – intensive care unit

IMU – intermediate care unit

KPC – Klebsiella pneumoniae carbapenemases

PDR – pandrug-resistant

PFGE – pulsed-field gel electrophoresis

SOI – severity of illness

STs – sequence types

VAP – ventilator-associated pneumonia

VIM – Verona integron-encoded metallo--lactamases

WGS – whole-genome sequencing
