# Supplementary material for: Clonal spread and environmental persistence of carbapenem-resistant high-risk Pseudomonas aeruginosa in critical-care units of a Chilean national referral center for burn and trauma patients (2022)
Source: Microb Cell. 2026 Jul 20;13:293–303. doi: 10.15698/mic2026.07.883 (PMC13389742; doi:10.15698/mic2026.07.883)
Supplement: Supplementary file 1 — . [file mic-13-293-s01.pdf]

Annex 1.

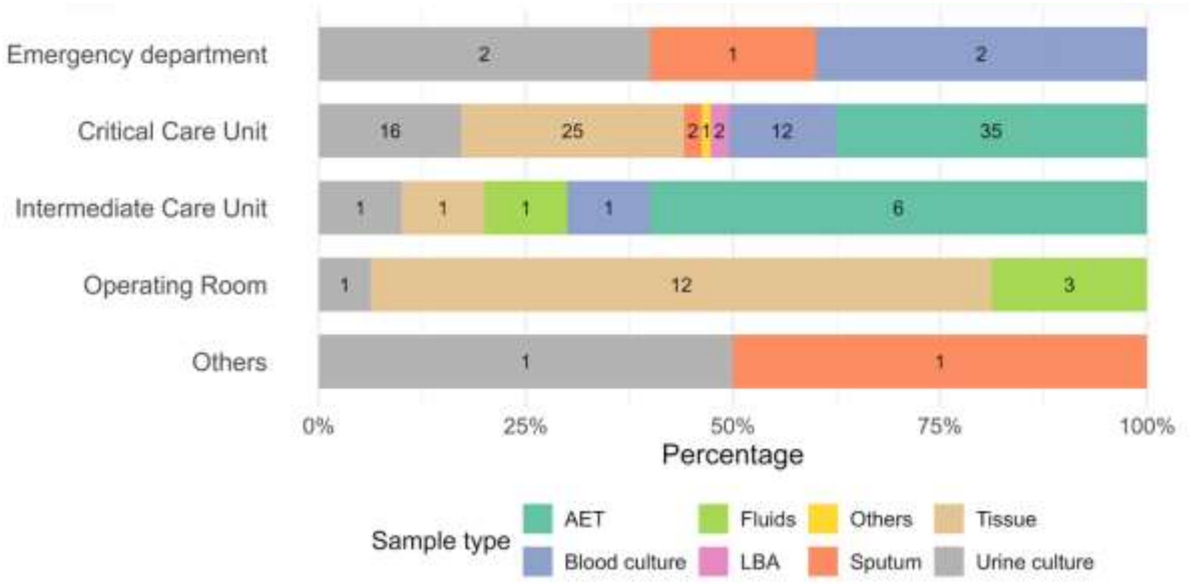

Figure S1. Distribution of samples by hospital department (HUAP). Graphical representation of the distribution of samples across the different hospital services of HUAP. The number of isolates per sample type is shown for each bar.
BAL: Bronchoalveolar lavage.

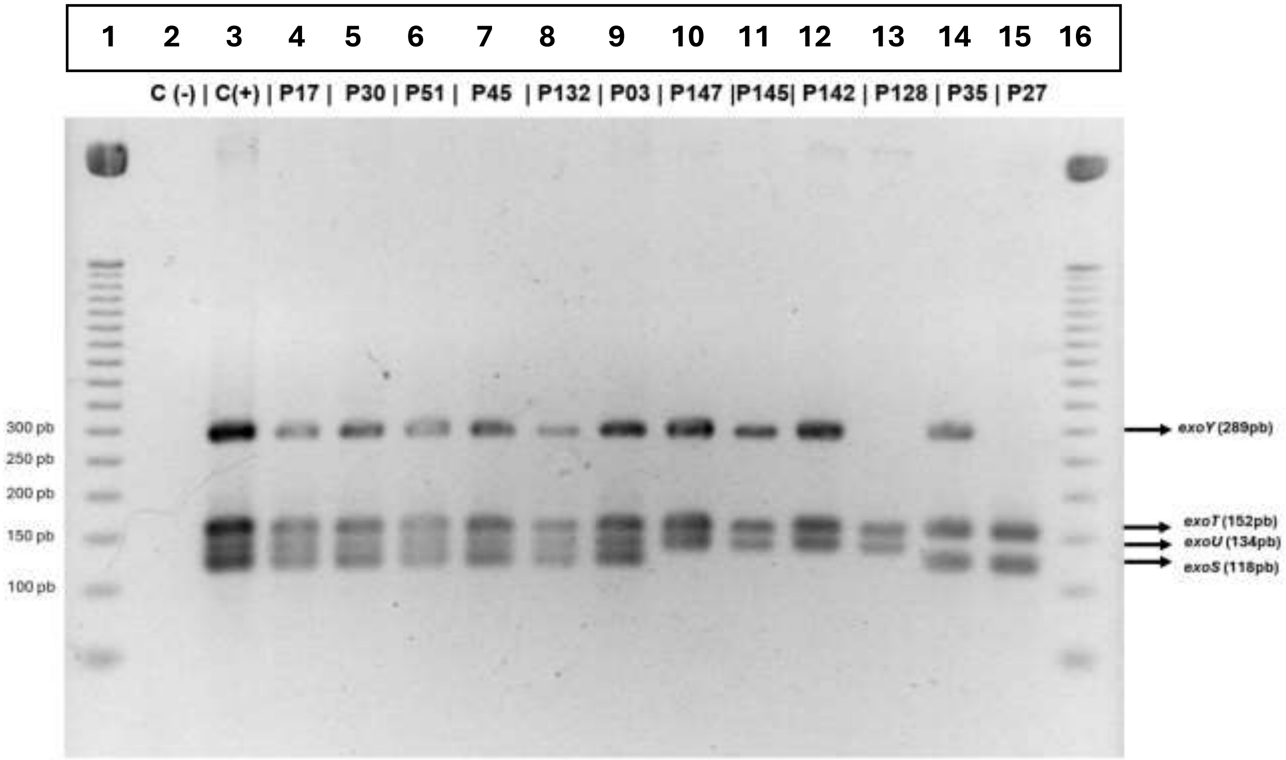

Figure S2. Detection of *exoY, exoT, exoU,* and *exoS* genes by multiplex PCR. The different genotypes detected for the *exoY, exoT, exoU,* and *exoS* genes are shown. The first and last lanes correspond to the 50 bp DNA ladder. Lane 2 shows the negative control, and lane 3 corresponds to the positive control containing the *Pseudomonas aeruginosa* ATCC 27853 and *P. aeruginosa* PA14 strains. Lanes 4-9 show the six isolates carrying the *exoY*$^{+}$*, exoT*$^{+}$*, exoU*$^{+}$*,* and *exoS*$^{+}$ genotype. Lanes 10-12 correspond to isolates with *exoY*$^{+}$*, exoT*$^{+}$*,* and *exoU*$^{+}$ genotype. Lane 13 shows one isolate with the *exoT*$^{+}$ and *exoU*$^{+}$ genotype, lane 14 one isolate with the *exoY*$^{+}$*, exoT*$^{+}$*,* and *exoS*$^{+}$, and lane 15 one isolate with the *exoT*$^{+}$ and *exoS*$^{+}$.

Table S1. Annual hospital activity and burden of *Pseudomonas aeruginosa* at HUAP (2019–2022).

Year	Total Patients hospitalized + emergency	Total Hospitalized Patients	Patients Requiring Invasive Mechanical Ventilation	Positive *Pseudomonas aeruginosa* culture
2019	ND	8.358	605	586
2020	10.778	8.780	1.097	863
2021	12.934	10.472	1.394	946
2022	14.010	10.682	951	798

ND: No data available.

Table S2. Characteristics of patients with multiple *Pseudomonas aeruginosa* isolates recovered during hospitalization.

Patient	Strain	Sample	Pulsogroup	Pulsotype	Resistance genotype (*blaKPC, blaNDM, blaVIM, blaOXA* y *blaIMP)*	Virulence factors genotype (exoenzymes)
1 (G.P.H)	P145	CAET	A	1	***blaVIM+***	*exoY+,exoT+,**exoU+***
	P34	BC	-	22	NEGATIVE	*exoY+,exoT+,exoS+*
2 (J.C.C)	P117	Tissue	O	43	NEGATIVE	*exoY+,exoT+,exoS+*
	P73	UC	O	44	NEGATIVE	*exoY+,exoT+,exoS+*
	P149	BC	J	24	***blaVIM+***	*exoY+,exoT+,exoS+*
3 (R.A.A)	P142	CAET	A	1	***blaVIM+***	*exoY+,exoT+,**exoU+***
	P52	CAET	Y	72	NEGATIVE	*exoY+,exoT+,exoS+*

Abbreviations: CAET, endotracheal aspirate culture; BC, blood culture; Tissue, tissue sample; UC, urine culture.

Table S3. Primers used for multiplex PCR of resistance genes.

Primer	DNA sequence (5’ a 3’)	Amplicon size	Reference
*blaKPC*_for	CGTCTAGTTCTGCTGTCTTG	798 bp	(Candan & Aksöz, 2015; Poirel et al., 2011)
*blaKPC*_rev	CTTGTCATCCTTGTTAGGCG		
*blaNDM*_for	GGTTTGGCGATCTGGTTTTC	621 bp	
*blaNDM*_rev	CGGAATGGCTCATCACGATC		
*blaOXA48*_for	GCGTGGTTAAGGATGAACAC	438 bp	
*blaOXA48*_rev	CATCAAGTTCAACCCAACCG		
*blaIMP*_for	GGAATAGAGTGGCTTAAYTCTC	232 bp	
*blaIMP*_rev	GGTTTAAYAAAACAACCACC		
*blaVIM*_for	GATGGTGTTTGGTCGCATA	390 bp	
*blaVIM*_rev	CGAATGCGCAGCACCAG		

Table S4. Primers used for multiplex PCR of virulence factors.

Primer	DNA sequence (5’ a 3’)	Amplicon size	Reference
exoS for	GCGAGGTCAGCAGAGTATCG	118 bp	(Ajayi et al., 2003)
exoS rev	TTCGGCGTCACTGTGGATGC		
exoT for	AATCGCCGTCCAACTGCATGCG	152 bp	
exoT rev	TGTTCGCCGAGGTACTGCTC		
exoU for	CCGTTGTGGTGCCGTTGAAG	134 bp	
exoU rev	CCAGATGTTCACCGACTCGC		
exoY for	CGGATTCTATGGCAGGGAGG	289 bp	
exoY rev	GCCCTTGATGCACTCGACCA		
algD for	CGTCTGCCGCGAGATCGGCT	313 bp	(Faraji et al., 2016)
algD rev	GACCTCGACGGTCTTGCGGA		
lasB for	GGAATGAACGAAGCGTTCTCCGAC	284 bp	
lasB rev	TTGGCGTCGACGAACACCTCG		
plcN for	TCCGTTATCGCAACCAGCCCTACG	481 bp	
plcN rev	TCGCTGTCGAGCAGGTCGAAC		
toxA for	GGTAACCAGCTCAGCCACAT	352 bp	Park and Koo (2022), Bogiel et al. (2021), and Lanotte et al. (2004)
toxA rev	TGATGTCCAGGTCATGCTTC		
plcH for	GAAGCCATGGGCTACTTCAA	307 bp	
plcH rev	AGAGTGACGAGGAGCGGTAG		

Table S5. PCR protocol for resistance and virulence genes.

	T°	Cycles
blaKPC, blaNDM, blaOXA-48, blaIMP, blaVIM		
**Initial denaturation**	95°C for 2 minutes	35
**Denaturation**	95°C for 1 minute	
**Annealing**	58°C for 1 minute	
**Extension**	72°C for 1 minute	
**Final extension**	72°C for 5 minutes	
exoS, exoT, exoU, exoY		
**Initial denaturation**	94°C for 2 minutes	30
**Denaturation**	95°C for 30 seconds	
**Annealing**	58°C for 30 seconds	
**Extension**	68°C for 1 minute	
**Final extension**	68°C for 7 minutes	
algD, lasB, plcN		
**Initial denaturation**	95°C for 5 minutes	30
**Denaturation**	94°C for 1 minute	
**Annealing**	60°C for 1,5 minutes	
**Extension**	72°C for 1 minute	
**Final extension**	72°C for 7 minutes	
toxA, plcH		
**Initial denaturation**	94°C for 2 minutes	35
**Denaturation**	94°C for 30 seconds	
**Annealing**	55°C for 30 seconds	
**Extension**	72°C for 30 seconds	
**Final extension**	72°C for 7 minutes	

Annex 2. Ethics Committee and HUAP Director Authorization Letters.

MINISTERIO DE SALUD
SERVICIO DE SALUD M. CENTRAL
COMITÉ ETICO CIENTIFICO
Teléfono: 25746958.5743520
RMR/ MCVS
.(Acta N° 69-10 N°350 /2023)

CERTIFICADO

DR. EMILIANO SOTO ROMO, en calidad de presidente del Comité Ético-Científico (CEC), del Servicio de Salud Metropolitano Central, constituido por resolución exenta N°1303 de fecha 26 de septiembre del 2002 de la Dirección de dicho Servicio y Acreditado por la SEREMI-RM mediante resolución N° 048975 del 30 de Julio del 2015 y re Acreditado mediante Resolución exenta por la SEREMI-RM el 22 de diciembre de 2021, certifica que en reunión expedita del 21 de noviembre del 2023, el CEC SSMC analiza las modificaciones solicitadas al Protocolo de investigación: **"Caracterización molecular de Pseudomonas aeruginosa resistente a carbapenémicos causante de infecciones nosocomiales en servicios críticos del Hospital de Urgencia Asistencia Pública (HUAP) durante el periodo 2022-2023",** cuya investigadora principal es la **TM. Camila Ibarra** del Hospital de Urgencia Asistencia Pública y cuyo subinvestigador es el **PhD. Msc. Roberto Vidal A.**, Profesor titular programa de Microbiología y Micología, ICBM, Facultad de Medicina, Universidad de Chile.

Se acusa recibo de los siguientes documentos:

- Proyecto de investigación Versión 2.0, Fecha 06-11-2023
- Ficha de protocolo
- Autorización de jefatura laboratorio clínico TM. Daniela Gutierrez M.
- Carta de compromiso de los investigadores
- Ausencia de conflicto de interés de los investigadores
- Carta de solicitud de exención del consentimiento informado versión del 09 de noviembre de 2023
- Pantallazo base de datos
- Carta jefe unidad de IAAS HUAP. Dr. Claudio Vargas R.

El CEC-SSMC decide Aprobar:

Caracterización molecular de Pseudomonas aeruginosa resistente a carbapenémicos causante de infecciones nosocomiales en servicios críticos del Hospital de Urgencia Asistencia Pública (HUAP) durante el periodo 2022-2023", y la Exención al Consentimiento Informado.

Se recuerda al investigador que:

Una vez aprobado el estudio por parte del CEC-SSMC, el investigador tiene la obligación de informar y solicitar la autorización para llevar a cabo el protocolo de investigación al director del establecimiento.

La validación ética dura un año y de acuerdo con la actual normativa, el investigador tiene la responsabilidad en comunicar al CEC, todo lo relacionado con el estudio: modificaciones, enmiendas, eventos adversos, desviaciones, suspensión del estudio, término del estudio, cierre del sitio, etc.
Para los estudios que duren menos de un año, los investigadores tienen el compromiso de hacer llegar el informe de término de la investigación.

Se recuerda que los eventos adversos debe hacerlos llegar también al Instituto de Salud Pública (ISP).

El CEC-SSMC tiene la facultad de realizar visitas en terreno a los sitios de investigación, como parte del seguimiento de los estudios. De acuerdo con la normativa vigente, dichas visitas se avisarán con al menos 48 horas de antelación.

Para ingresar las nuevas versiones de documentos, se solicita a los investigadores hacer llegar:
Carta conductora dirigida al presidente del Comité, solicitando la aprobación, (traer en duplicado)

Se adjunta: copia de carta enviada por la Investigadora, firmada, fechada y timbrada
Nº 330 de recepción.

La reunión expedita, contó con la presencia de la Sra. Marlene Valenzuela Saavedra, y el Dr. Emiliano Soto Romo.

SERVICIO DE ... METROPOLITANO CENTRAL — COMITÉ ETICO-CIENTIFICO

EU MARLENE VALENZUELA SAAVEDRA
SECRETARIA EJECUTIVA CEC S.S.M.C

DR. EMILIANO SOTO ROMO
PRESIDENTE CEC S.S.M.C

CARTA AUTORIZACIÓN

Revisado el certificado de aprobación del Comité Ético Científico del SSMC para el proyecto de investigación titulado "**CARACTERIZACIÓN MOLECULAR DE PSEUDOMONAS AERUGINOSA RESISTENTE A CARBAPENÉMICOS CAUSANTE DE INFECCIONES NOSOCOMIALES EN SERVICIOS CRÍTICOS DEL HOSPITAL DE URGENCIA ASISTENCIA PÚBLICA (HUAP) DURANTE EL PERÍODO 2022- 2023"**, cuyo investigador principal es la TM. Camila Ibarra Castro, profesional del Laboratorio Clínico HUAP, y en donde el rol de subinvestigador lo tendrá el Dr. Roberto Vidal, profesor titular del programa de Microbiología y Micología de la Facultad de Medicina en la Universidad de Chile, indico mediante este documento la **AUTORIZACIÓN** de la realización del estudio en este establecimiento.

Sin otro particular,

Atentamente

HOSPITAL DE URGENCIA ASISTENCIA PÚBLICA DIRECTOR

DR. JORGE IBAÑEZ P.

DIRECTOR (S)

HOSPITAL DE URGENCIA ASISTENCIA PÚBLICA

En Santiago, 05 de diciembre de 2023.

c.c. - Unidad de Investigación HUAP
- TM. Camila Ibarra
